# Accurate real-time obstacle detection of coal mine driverless electric locomotive based on ODEL-YOLOv5s

**DOI:** 10.1038/s41598-023-44746-8

**Published:** 2023-10-14

**Authors:** Tun Yang, Shuang Wang, Jiale Tong, Wenshan Wang

**Affiliations:** 1 State Key Laboratory of Mining Response and Disaster Prevention and Control in Deep Coal Mines, Anhui University of Science and Technology, Huainan, 232001 China; 2 School of Mechanical Engineering, Anhui University of Science and Technology, Huainan, 232001 China; 3 Collaborative Innovation Center for Mining Intelligent Technology and Equipment, Huainan, 232001 China

**Keywords:** Mechanical engineering, Computer science

## Abstract

The accurate identification and real-time detection of obstacles have been considered the premise to ensure the safe operation of coal mine driverless electric locomotives. The harsh coal mine roadway environment leads to low detection accuracy of obstacles based on traditional detection methods such as LiDAR and machine learning, and these traditional obstacle detection methods lead to slower detection speeds due to excessive computational reasoning. To address the above-mentioned problems, we propose a deep learning-based ODEL-YOLOv5s detection model based on the conventional YOLOv5s. In this work, several data augmentation methods are introduced to increase the diversity of obstacle features in the dataset images. An attention mechanism is introduced to the neck of the model to improve the focus of the model on obstacle features. The three-scale prediction of the model is increased to a four-scale prediction to improve the detection ability of the model for small obstacles. We also optimize the localization loss function and non-maximum suppression method of the model to improve the regression accuracy and reduce the redundancy of the prediction boxes. The experimental results show that the mean average precision (mAP) of the proposed ODEL-YOLOv5s model is increased from 95.2 to 98.9% compared to the conventional YOLOv5s, the average precision of small obstacle rock is increased from 89.2 to 97.9%, the detection speed of the model is 60.2 FPS, and it has better detection performance compared with other detection models, which can provide technical support for obstacle identification and real-time detection of coal mine driverless electric locomotives.

## Introduction

The coal mine rail electric locomotive, one of the major pieces of equipment in coal mine production, undertakes the transportation tasks (e.g., underground operators and gangue), which is a vital link to determining the efficient production of coal mines^1^. The driverless technology of coal mine rail electric locomotives has aroused wide attention as coal mine intelligence leaps forward^2^. The driverless technology of electric locomotives is capable of avoiding electric locomotive collisions, derailments, and other accidents caused by the poor coal mine roadway environment and the uncertainty of manual driving. Moreover, this technology is capable of achieving the purpose of reducing personnel and increasing efficiency, thus having significant economic benefits.

The driverless technology of electric locomotives is essentially designed to identify and detect obstacles that may appear in front of the locomotive for autonomous obstacle avoidance. The existing research on the driverless technology of electric locomotives in coal mines worldwide remains in the preliminary stage. The obstacle detection technology in complex coal mine roadway environments is not mature. A few domestic coal mines realize the obstacle detection of electric locomotives based on LiDAR technology and, at the same time, realize electric locomotives that are driverless with remote monitoring and an intelligent dispatching system. However, the processing process based on LiDAR detection is complicated and vulnerable to environmental effects^3,4^. Narrow coal mine roadway space and uneven roadway walls will make the data collected by LiDAR have more noise, thus resulting in an unsatisfactory detection effect. In contrast, image processing techniques-based deep learning target detection algorithms automatically learn image features based on convolutional neural networks (CNN), which have been gradually used in extensive target detection tasks in coal mines for their higher detection accuracy and faster detection speed. Xu et al. proposed a CAP-YOLO algorithm for intelligent monitoring of pedestrians in coal mines through the lightweight improvement of the YOLOv3 algorithm, thus effectively increasing the reasoning speed of the algorithm and detecting pedestrians in real-time^5^. Liu et al. applied the optimized YOLOv4 model to the intelligent separation task of coal and gangue, thus increasing the recognition accuracy and speed^6^. Cai et al. proposed a deep learning-based detection algorithm for granularity detection and identification of mine-dumped materials, aggregates, and clays with good detection results^7^. Li et al. proposed a TMRC-SSD algorithm to identify and detect gangue, bolt, stick, and other impurities mixed in raw coal, thus ensuring the safe production and mining efficiency of coal^8^. It can be seen that the above scholars have improved the deep learning target detection algorithms to improve the detection accuracy and speed of the targets in different scenarios to varying degrees and realize accurate real-time detection. Therefore, applying deep learning detection algorithms to the obstacle detection of driverless electric locomotives will be helpful in improving the detection accuracy of obstacles and guaranteeing real-time detection, which is of great significance for improving the safe operation of driverless electric locomotives.

Deep learning target detection algorithms are classified into two types (including two-stage and one-stage), among which two-stage algorithms include FAST-RCNN^9^, MASK-RCNN^10^, etc., and one-stage detection algorithms include Single Shot Detector (SSD)^11^ and You Only Look Once (YOLO) series^12–15^, and so on. Two-stage detection algorithms have a deep network structure and a large amount of computation, resulting in slower detection speeds for these algorithms, while one-stage algorithms have a relatively simple network structure and fast detection speeds, making them suitable for scenarios that require high detection speeds. Since the two-stage detection algorithm cannot meet real-time detection^16–18^, this study adopts YOLOv5s, which has a faster detection speed, as the detection model. The model is optimized according to the characteristics of insufficient light, partial occlusion, and a large number of small obstacles in the coal mine roadway to improve the detection accuracy of the model, and the ODEL-YOLOv5s model is proposed. The main contributions of this study are as follows:

1. Mixup, Cutmix, Cutout, and Mosaic data augmentation methods are introduced to expand the dataset and improve the diversity of obstacle features.
2. The Convolutional Block Attention Module (CBAM) is introduced to the neck of the model to focus on interesting features and increase the receptive field of the model.
3. A small target prediction layer is added to the model to enhance the detection ability of the model for small obstacles.
4. The localization loss function and non-maximum suppression method of the model are optimized to improve the regression and reduce the redundancy of the prediction boxes.

### YOLOv5 model description

#### Detection principle of YOLO algorithm

The YOLO algorithm is capable of simplifying the problem of target detection into a regression problem^19^. Its core idea is to take the whole image as the input of the model, extract the input image features on CNN multi-scale, further fuse the extracted features, and then send the image features to the prediction layer for bounding box regression and category probability prediction. The YOLO algorithm can divide the input image evenly into N × N squares. B bounding boxes, and the confidence scores of the above bounding boxes will be predicted in the respective square. If the center of an obstacle in the image is located in a square, this square is ultimately responsible for the prediction of such an obstacle^20^.

#### YOLOv5 model structure

**Figure 1:**
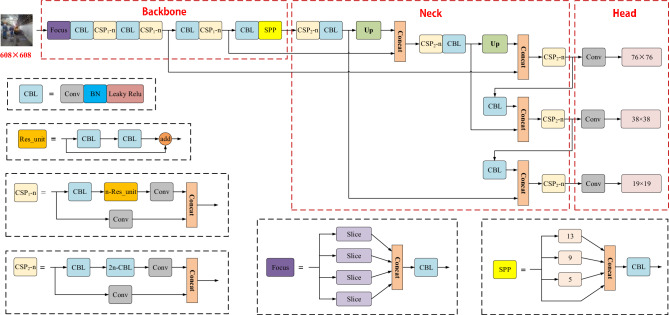
Schematic diagram of YOLOv5 model structure.

There are four versions of the YOLOv5 model, i.e., YOLOv5s, YOLOv5m, YOLOv5l, and YOLOv5x. To be specific, the YOLOv5s model is the lightest version in the YOLOv5 series; the other versions are deeper and wider based on the YOLOv5s model. As depicted in Fig. 1, the YOLOv5 model comprises three parts (including the Backbone, Neck, and Head). In the Backbone part, the Focus module is first adopted to slice the input image. Subsequently, the image is concatenated, and then a series of convolution operations are performed, thus effectively reducing the calculation cost and increasing the speed of extracting features. In the Neck part, the YOLOv5 model adopts Spatial Pyramid Pooling (SPP)^21^ and Path Aggregation Network (PANet)^22^. The SPP module is capable of extracting fixed-size feature vectors from different-scale feature maps to solve the problem of different sizes of input images, thus effectively increasing the receptive field of the model. Under the PANet module, path expansion and aggregation are conducted to enhance the bottom-up path, thus making bottom-level information easier to propagate and shortening the information path between low-level and high-level features. The YOLOv5 model draws lessons from Cross Stage Partial Network (CSPNet)^23^ while designing CSP_1_-n and CSP_2_-n modules in accordance with whether there are residual components. The CSP_1_-n is applied to the Backbone of the model, and the CSP_2_-n is applied to the Neck of the model. In the Head part, the YOLOv3 detection head is employed by the YOLOv5 model for multi-scale prediction.

#### Loss function

The loss function is the difference between the predicted value and the true value of the model. The smaller the value of the loss function, the higher the prediction accuracy of the model will be and the better the classification effect will be. The loss of the YOLOv5 model comprises three parts, namely classification loss, confidence loss, and localization loss.

**Figure 2:**
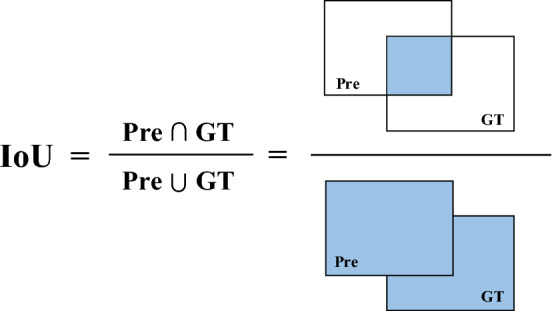
Schematic diagram of IoU structure.

The IoU loss function (L_IoU_) is usually employed as the localization loss of the model in the field of target detection, i.e., the bounding box regression loss. As depicted in Fig. 2, the IoU represents the ratio of the overlapping area of the prediction box (Pre) and ground truth box (GT) to the area of union of the two boxes, which is adopted to measure the overlap ratio between the Pre and the manually marked GT; the larger the value of the IoU, the more accurate the predicted obstacle area will be.

The GIoU loss function (L_GIoU_) serves as the bounding box regression loss in the conventional YOLOv5 model. The L_GIoU_ considers the minimum closure rectangular area of the Pre and the GT based on the L_IoU_ to avoid the problem that the Pre and the GT have no overlapping area, thus making the value of the IoU approach zero and causing the model training to fail to converge. The L_GIoU_ calculation equation is presented as follows, where *A*_*c*_ denotes the minimum closure rectangular area of the Pre and the GT, and *U* expresses the union area of the Pre and the GT.

1 $$ {\text{L}}_{\text{GIoU}} = 1 - {\text{GIoU}} $$

2 $$ {\text{GIoU}} = {\text{IoU}} - \frac{{A_{c} - U}}{{A_{c} }} $$

## Methods

Compared with the other three versions of YOLOv5 models, the YOLOv5s model is small in size, low in computation, and faster in detection, thus making it suitable for deployment on embedded devices with limited resources^24^. In this study, the YOLOv5s model is employed as the research object, and the detection performance of the model in the harsh coal mine roadway environment is enhanced by different improvement measures.

### Data augmentation

**Figure 3:**
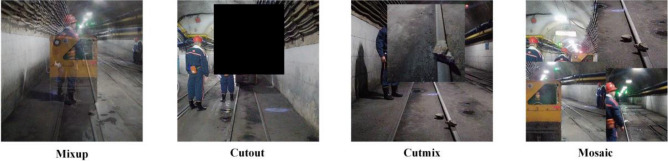
Several data augmentation methods.

Data augmentation aims to expand the dataset and enrich its characteristics so that the model is more robust to images obtained in different environments. Data augmentation methods are primarily divided into two types, i.e., photometric distortion-based and geometric distortion-based. In this study, the saturation, exposure, and hue of the dataset images are adjusted for the photometric distortion data augmentation method. For the geometric distortion data augmentation method, Mixup^25^, Cutout, Cutmix^26^, and Mosaic data augmentation are also adopted to process the images, besides cropping, shearing, rotating, and flipping the images. To be specific, the Mixup is to randomly mix and blur two samples in the dataset. The Cutout randomly removes some areas in a sample and fills them with zero-pixel values. The Cutmix randomly removes some regions of one sample and then randomly fills in the regional pixel values of other samples in the dataset. The Mosaic data augmentation aims to randomly select four pictures from the dataset for random cutting, scaling, and splicing, thus effectively expanding the characteristics of small targets and increasing the robustness of the model. Figure 3 presents the data augmentation effect of the Mixup, Cutout, Cutmix, and Mosaic.

### Introduce CBAM attention mechanism

**Figure 4:**
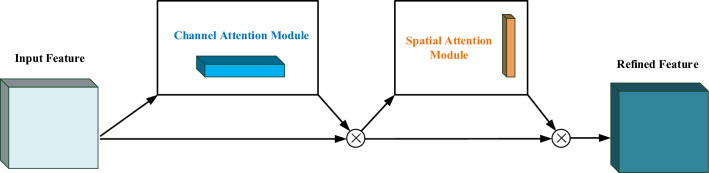
Schematic diagram of CBAM structure.

The attention mechanism helps the model locate the information of interest from the images and select the information more critical to the current task targets from the numerous sources of information. The Convolutional Block Attention Module (CBAM)^27^ refers to a lightweight attention module that can be integrated into any CNN architecture and trained end-to-end with basic CNN. Given an intermediate feature map, CBAM sequentially extracts attention features along the two dimensions of channel and space and then combines the extracted attention feature map with the original feature map to refine the adaptive features. These features are employed as the input for the next convolution layer. In this study, the CBAM attention mechanism is introduced into the conventional YOLOv5s model, so the model can focus more on the obstacle region in the images at the training stage and increase the detection accuracy of the model. The CBAM structure is illustrated in Fig. 4.

### Add small target prediction layer

The conventional YOLOv5s model adopts three scales of detection layers for target perdition, and for the input 608 × 608 image, the feature map sizes on the three detection layers are 19 × 19, 38 × 38, and 76 × 76. The downsampling multiple of image features extracted by the YOLOv5s model is large, thus making it difficult for the deep feature map to learn small target feature information, which causes missed and inaccurate detection of small obstacles in conventional YOLOv5s. Accordingly, a small target prediction layer is introduced to the conventional YOLOv5s model, and the shallow feature map of 152 × 152 scale in Backbone and the deep feature map of 152 × 152 scale in Neck after feature fusion are concatenated to increase the detection effect of the model for small obstacles.

### Improve IoU loss function

The L_GIoU_ employed in the conventional YOLOv5s model can also measure the prediction results when no overlapping area exists between the Pre and the GT. However, the L_GIoU_ has certain problems. In accordance with Eqs. (1)–(2), when there is an inclusion relationship between the Pre and the GT, the union area *U* of the Pre and the GT is the same as their minimum closure rectangular area *A*_*c*_, and the GIoU is degenerated into the ordinary IoU. Thus, the relative positions of the Pre and the GT cannot be evaluated, causing slow convergence of the model in the training process.

To solve this problem, this study improves the L_GIoU_ to CIoU loss (L_CIoU_), and a power index *α* is added to the L_CIoU_ to obtain α-CIOU loss (L_α-CIoU_)^28^. The L_CIoU_ considers the overlapping area, center point distance, aspect ratio, and penalty term of the Pre and the GT at the same time, making the bounding box regression more stable and achieving faster convergence. The equation of the L_CIoU_ is expressed as Eq. (3), where *b* and *b*^gt^ denote the centers of the Pre and the GT, respectively; *ρ*(*b*, *b*^gt^) represents the center distance between the Pre and the GT; *c* expresses the diagonal length of the minimum closure area of the Pre and the GT; *β* represents a weight function; and *v* is adopted to measure the aspect ratio. The equation of *β* and *v* is expressed as Eqs. (4)–(5), where *w* and *h* are the width and height of the Pre, *w*^gt^ and *h*^gt^ are the width and height of the GT.

3 $$ {\text{L}}_{{{\text{CIoU}}}} = 1 - {\text{IoU}} + \left( {\frac{{\rho (b,b^{{{\text{gt}}}} )}}{c}} \right)^{2} + \beta \nu $$

4 $$ \beta = \frac{\nu }{{(1 - {\text{IoU}}) + \nu }} $$

5 $$ v = \frac{4}{{\pi^{2} }}\left( {\arctan \frac{{w^{{{\text{gt}}}} }}{{h^{{{\text{gt}}}} }} - \arctan \frac{w}{h}} \right)^{2} $$

By modulating the value of the *α* (*α* = 3 in this study), the detection layer of the model exhibits higher flexibility in bounding box regression, thus effectively increasing the accuracy of bounding box regression without additional training or reasoning time for the model. The calculation equation of the L_α-CIoU_ is expressed as Eq. (6).

6 $$ {\text{L}}_{\alpha - {\text{CIoU}}} = 1 - {\text{IoU}}^{\alpha } + \left( {\frac{{\rho (b,b^{\text{gt}} )}}{c}} \right)^{2\alpha } + \beta \nu $$

### Optimize the non-maximum suppression method

The conventional YOLOv5s model adopts weighted non-maximum suppression (NMS) to remove redundant prediction boxes and retain the box with the highest confidence in the prediction stage. Compared with the conventional NMS method, the weighted NMS method can effectively improve prediction accuracy by weighting and summing up the information in all prediction boxes. However, the weighted NMS method has high computational complexity and low computational efficiency. Moreover, in the practical application of the harsh coal mine roadway environment, when the obstacles are dense or partially blocked, the effect of using the weighted NMS method is not ideal. Therefore, this study adopts the Cluster-NMS method as the non-maximum suppression processing method of the ODEL-YOLOv5s model. The Cluster-NMS algorithm exhibits a fast reasoning speed and better suppression effect of redundant prediction boxes, and it will not excessively increase the number of iteration rounds with the increase in the number of detected targets, thus effectively reducing the time complexity^29^.

**Figure 5:**
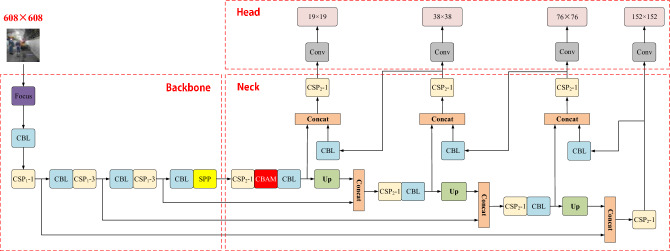
Schematic diagram of ODEL-YOLOv5s model structure.

The structure of the ODEL-YOLOv5s model proposed in this study is illustrated in Fig. 5. The introduced CBAM attention mechanism is added to the Neck of the model (as shown in the red box in Fig. 5). Second, the 76 × 76 feature map is upsampled by twofold at the end of the Neck, and subsequently concatenated with the feature map processed by the first CSP_1_ module of the Backbone, resulting in the addition of a new 152 × 152 prediction scale at the Head. The number of layers in the optimized YOLOv5s model is increased from the original 224 layers to 281 layers, and the weight size and reasoning time of the model are increased. The pseudocode for improving the model is shown in Table 1.

**Table 1:** Pseudocode of improving the YOLOv5s model.

**Algorithm 1: Improved YOLOv5s Algorithm**	
**Require:** Training dataset with obstacle images	
**Ensure:** Trained YOLOv5s model	
1: **Procedure: Data Preparation**	
2: Collect and annotate dataset for obstacle detection in coal mine roadways	
3: Split dataset into training, validation, and testing sets	
4:** Procedure: Data Augmentation**	
5: Apply various data augmentation techniques, including adjusting saturation, exposure, and hue of images	
6: Perform geometric distortion such as cropping, shearing, rotating, and flipping	
7: Introduce data augmentation methods like Mixup, Cutout, Cutmix, and Mosaic	
8:** Procedure: Build Detection Model**	
9: Use a backbone network (e.g., CSPDarknet53) to extract image features	
10: Fuse features in the neck network by combining shallow and deep feature maps	
11: Add a prediction layer in the head for detecting small obstacles by concatenating fused features with 152 × 152-scale shallow feature maps	
12:** Procedure: Introduce CBAM Attention Mechanism**	
13: Integrate Convolutional Block Attention Module (CBAM) into YOLOv5s model	
14: Extract attention features along channel and spatial dimensions using CBAM	
15: Combine attention-enhanced features with original feature maps for adaptive feature refinement	
16: **Procedure: Train the Model**	
17: Calculate loss using improved IoU loss function, such as α-CIoU loss:$L_{\alpha - {\text{CIoU}}} = 1 - {\text{IoU}}^{\alpha } + \left( {\frac{{\rho (b,b^{gt} )}}{c}} \right)^{2\alpha } + \beta \nu$	
18: Adopt Cluster-NMS as the non-maximum suppression processing method	
19: Update model parameters through backpropagation and optimization algorithms (e.g., stochastic gradient descent)	
20: Evaluate model performance on the validation set during training and make adjustments accordingly	
21: **Procedure: Model Evaluation and Testing**	
22: Evaluate trained YOLOv5s model using the testing set	
23: Compute metrics such as AP, mAP, recall, precision, etc	
24: Test the model on new images from coal mine roadways to assess obstacle detection performance	

## Experiment

### Making dataset

**Figure 6:**
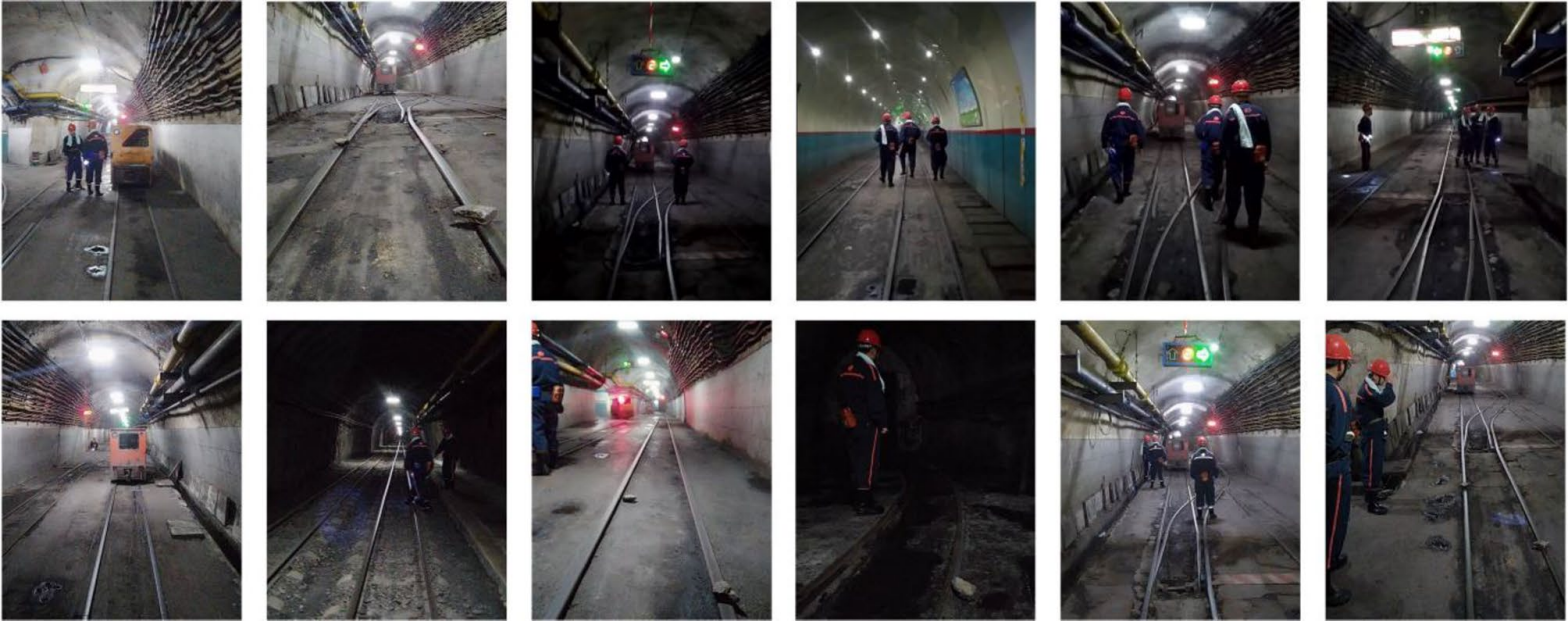
Partial dataset.

In this study, the obstacle detection image dataset required by the experiment is self-made, and the obstacles include miners, electric locomotives, and rocks. The special explosion-proof camera for coal mines is used to shoot on the transportation roadway about 750 m deep under Yuandian No. 1 coal mine in Huaibei City, Anhui Province. In order to increase the diversity of the dataset, the different positions, distances, angles, lighting conditions, and occlusion of obstacles were considered during shooting. A total of 2000 dataset images are obtained after post-processing, and some of the images of the dataset are shown in Fig. 6.

The LabelImg labeling tool is used to label the obstacles in the dataset images; that is, all the obstacles contained in the images were manually selected by the minimum enclosing rectangle and labeled with “Miner”, “E-L”, and “Rock”, respectively. Lastly, the labeled dataset is divided into training set and test set in a ratio of 3:1.

#### Model evaluation indicators

In this study, Precision, Recall, AP (average precision) and mAP (mean average precision), average detection speed, weight size and amount of calculation are employed as the indicators to evaluate model detection performance. To be specific, the Precision is adopted to measure whether the model is accurate in obstacle detection, and the Recall reflects whether the model is comprehensive in obstacle detection. The AP represents the average detection accuracy of the model for a class of obstacles, and the mAP refers to the average detection accuracy of the model for all kinds of obstacles. The calculation formulas are shown in (7)–(10), where TP denotes the number of positive samples correctly predicted by the model, FP represents the number of positive samples incorrectly predicted by the model, FN expresses the number of negative samples incorrectly predicted by the model, and *P*_*i*_ is the corresponding accuracy rate of *n* positive samples of a certain class of obstacles^30,31^.

7 $$ {\text{Precision}} = \frac{{{\text{TP}}}}{{\text{TP + FP}}} $$

8 $$ {\text{Recall}} = \frac{{{\text{TP}}}}{{\text{TP + FN}}} $$

9 $$ {\text{AP}} = \frac{1}{n}\sum\limits_{i = 1}^{n} {P_{i} } = \frac{{P_{1} + P_{2} + \ldots + P_{n} }}{n} $$

10 $$ {\text{mAP}} = \frac{{\sum\limits_{i = 1}^{n} {{\text{AP}}_{i} } }}{n} $$

#### Model training

The CPU of the computer employed in this experiment is an AMD Ryzen 7 5800X, and the main frequency is 3.8 GHz. The GPU is an NVIDIA GeForce RTX 3060 with 12 GB of video memory. The computer uses the Ubuntu 18.04 operating system, equipped with Cuda 11.0 and Cudnn 8.04 for GPU acceleration. Furthermore, the detection model is built on Python 1.7 and Python 3.6.

Before the training of the model, the training parameters of the configuration file are adjusted to yield the optimal model. The input image size is set to 608 × 608, the iteration batch size is 16, the momentum is 0.937, the decay is 0.0005, the learning rate is 0.1, and the number of iterations is set to 300.

**Figure 7:**
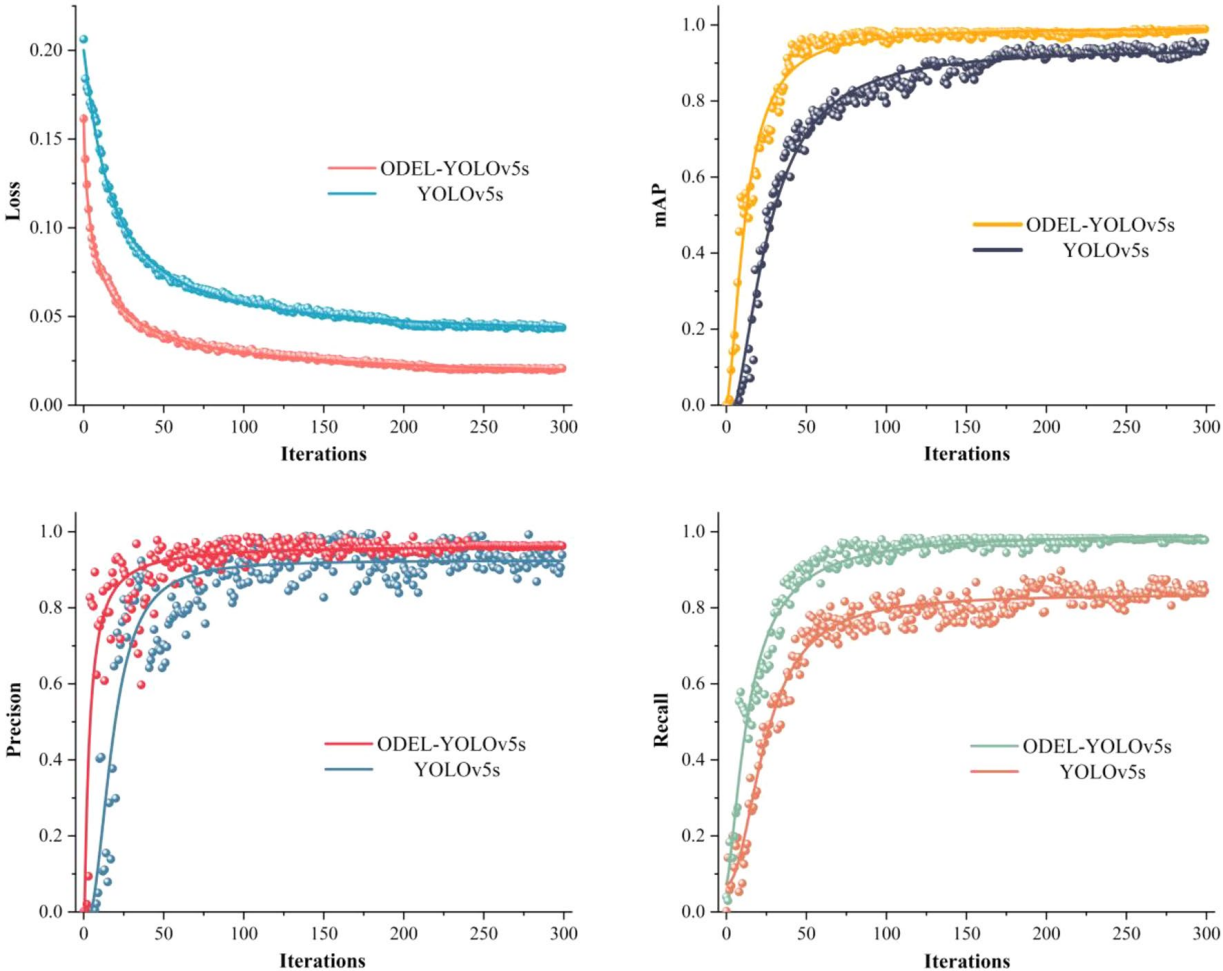
Curve changes before and after model improvement.

The training curve changes of the ODEL-YOLOv5s model and the conventional YOLOv5s model are presented in Fig. 7. The ODEL-YOLOv5s model has more stable curve changes, smaller loss values, and higher Precision, Recall, and mAP compared with the conventional YOLOv5s model.

#### Ablation experiment

In this study, a variety of improvement measures are adopted to enhance the detection performance of the YOLOv5s model for obstacles in the harsh coal mine roadway environment. In order to further verify the effectiveness of each improvement measure, the effect of each improvement measure is analyzed on the detection performance of the model through ablation experiments, and the experimental results are shown in Table 2.

**Table 2:** Ablation Experiment.

Model	Measures					AP (%)			mAP (%)	FPS
	Data augmentation	CBAM	4-Pre	α-CIoU	Cluster-NMS	Miner	E-L	Rock		
A						99.1	97.4	89.2	95.2	76.4
B	√					99.1	97.8	91.6	96.2	77.2
C	√	√				99.0	98.4	93.4	96.9	71.5
D	√	√	√			98.9	98.8	96.1	97.9	58.6
E	√	√	√	√		99.4	99.2	96.2	98.3	58.6
F	√	√	√	√	√	**99.5**	**99.4**	**97.9**	**98.9**	**60.2**

Significant values are bold.

Model A is the YOLOv5s model without any improvement measures, and its mAP is 95.2%; the AP of small obstacle rock accounts for 89.2%; and the average detection speed reaches 76.4 FPS.

Model B performs data augmentation processing based on model A. Compared with model A, the mAP of model B is increased by 1.0%, the AP of small obstacle rock is increased by 2.4%, and the average detection speed is increased by 0.8 FPS.

Model C adds the CBAM attention mechanism based on model B. Compared with model B, the mAP of model C is increased by 0.7%, and the AP of small obstacle rock is increased by 1.8%. The addition of CBAM slightly increases the amount of calculation in the model, resulting in its average detection speed falling to 71.5 FPS.

Model D adds a small target detection layer based on model C, so the model is improved from the original three-scale prediction (3-pre) to four-scale prediction (4-pre). Compared with model C, the mAP of model D is increased by 1.0%, and the AP of small obstacle rock is increased by 2.7%. Due to the increase in the number of layers and reasoning time of the model, the average detection speed of the model is reduced to 58.6 FPS.

Model E improves the loss function based on model D. Compared with model D, the mAP of model E is increased by 0.4%, the AP of small obstacle rock is increased by 0.1%, and the average detection speed does not change.

Model F uses Cluster-NMS based on model E. Compared with model E, the mAP of model F is increased by 0.6%, the AP of small obstacle rock is increased by 1.7%, and the average detection speed is increased by 1.6 FPS. Model F integrates all the improvement measures in this study, and each improvement measure improves the detection accuracy of the model to varying degrees.

## Results and discussion

To visualize the difference in detection results between ODEL-YOLOv5s and other mainstream target detection algorithms, these algorithms are trained and tested based on the same self-made dataset, and the detection results are listed in Table 3: The large number of computations (GFLOPs) in the YOLOv3 and YOLOv4 algorithms increases the inference time of the algorithms, resulting in an average detection speed of only 26.1 FPS and 20.5 FPS for YOLOv3 and YOLOv4, respectively, which is difficult to meet the needs of real-time detection in a harsh coal mine roadway environment. The average detection speed of YOLOV3-Tiny and YOLOV4-Tiny is 162.8 FPS and 156.2 FPS, respectively, but due to the simpler network structure of these two algorithms, they have limited ability to extract image features during the training process, resulting in poor detection of small obstacle rock. The mAP of YOLOv5x, YOLOv5l, and YOLOv5m all reach above 96%, but the weight size and calculation of the model are large, and the requirements for hardware equipment are high. For the novel versions of YOLOv6 and YOLOv7, the mAP of YOLOv6 is 92.9%, which is lower than the YOLOv5 series algorithms, the average detection speed of YOLOv6 is 33.7 FPS lower than YOLOv5s, and the weight size of the algorithms as well as the amount of computation is higher than YOLOv5s. The AP of YOLOv7 reaches 98.6% and 99.1% for large obstacle miner as well as electric locomotive, respectively, but the detection accuracy for small obstacle rock is only 71.3%, suggesting that the YOLOv7 is poor at detecting small obstacles. The mAP of the ODEL-YOLOv5s model proposed in this study reaches 98.9%, and the AP of small obstacle rock reaches 97.9%, which are 3.7% and 8.7% higher than the conventional YOLOv5s model, respectively. Compared with the conventional YOLOv5s model, the weight size of the ODEL-YOLOv5s model increases by 1.5 Mb, and the calculation amount increases by 11.3 GFLOPs, which are 15.9 Mb and 27.6 GFLOPs, respectively, but it is still at a low level compared with other algorithms in Table 3. Compared with the conventional YOLOv5s model, the average detection speed of the ODEL-YOLOv5s model is reduced by 16.2 FPS to 60.2 FPS, but it still meets the requirements of real-time detection. In summary, the ODEL-YOLOv5s model has higher average detection accuracy and better detection capability for small targets under the condition of ensuring real-time detection, and the small size of the model makes it easy to be deployed and used in mobile and embedded devices. The ODEL-YOLOv5s model proposed in this study has better detection performance than other algorithms.

**Table 3:** Comparative experimental results.

Model	AP (%)			mAP (%)	FPS	Weight Size (Mb)	GFLOPs
	Miner	E-L	Rock				
YOLOv3	97.3	94.3	80.7	90.8	26.1	246.3	139.5
YOLOv3-tiny	96.3	95.8	76.9	89.7	162.8	34.7	5.5
YOLOv4	98.2	98.2	85.2	93.9	20.5	256.1	127.3
YOLOv4-tiny	97.3	96.9	85.5	93.2	156.2	23.5	14.5
YOLOv5x	99.3	98.3	92.3	96.6	41.3	175.1	217.1
YOLOv5l	99.6	98.5	90.8	96.3	50.1	93.7	114.1
YOLOv5m	99.5	97.0	91.8	96.1	62.8	42.5	50.3
YOLOv5s	99.1	97.4	89.2	95.2	76.4	14.4	16.3
YOLOv6	99.9	96.5	82.2	92.9	42.7	38.0	44.1
YOLOv7	98.6	99.1	71.3	89.7	65.5	19.0	26.5
ODEL-YOLOv5s	99.5	99.4	97.9	98.9	60.2	15.9	27.6

**Figure 8:**
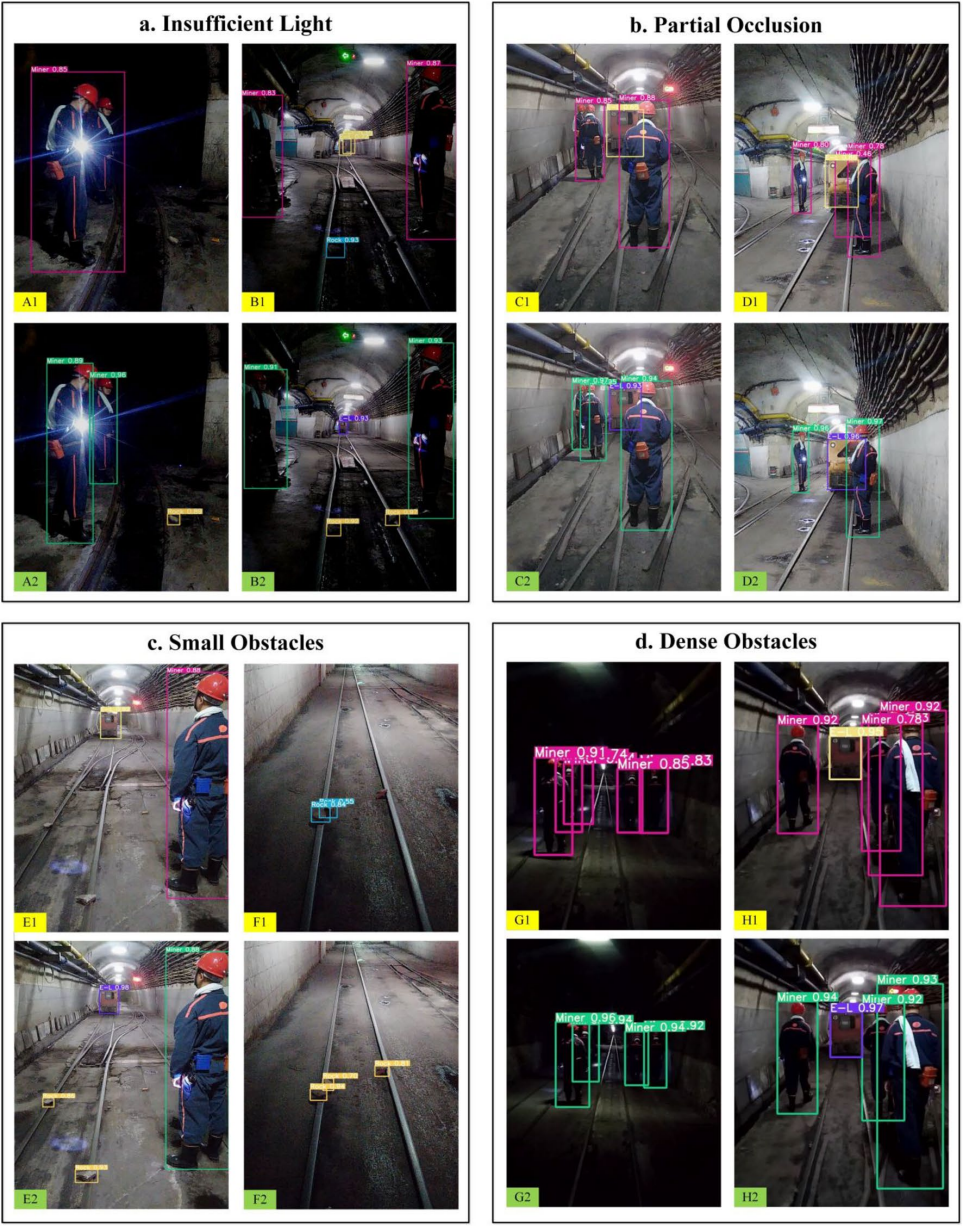
Comparison of test results.

In order to verify the detection effect of the ODEL-YOLOv5s model in real coal mine roadway scenarios, the detection results of the conventional YOLOv5s model and the ODEL-YOLOv5s model are compared and analyzed. The comparison of detection results between the two models is illustrated in Fig. 8, in which the image numbers A1-H1 represent the detection results of the conventional YOLOv5s model and A2-H2 are the detection results of the ODEL-YOLOv5s model. The comparison is carried out using the environmental scenarios of insufficient light, partial occlusion, small obstacles, and dense obstacles.

For the detection effect of the conventional YOLOv5s model, the obstacles of the miner and rock are missed in an environment with insufficient light, as shown in images A1-B1. As depicted in images C1-D1, the partially blocked miner is missed in image C1, and the partially blocked electric locomotive is mistakenly detected as a miner in image D1. As depicted in images E1-F1, missed detection occurs in the images of multiple small obstacle rocks. According to the images G1-H1, a redundant prediction box appears in the detection of the miner in the case of relatively dense obstacles.

For the detection effect of the ODEL-YOLOv5s model, as shown in images A2-H2. Due to the specific improvements made to the model according to the environmental characteristics of the coal mine roadway in this study, the detection ability of the model for obstacles is enhanced. Therefore, the obstacles can be detected accurately in the different environmental scenarios in which they are located. The comparative detection results in Fig. 8 show that compared with the conventional YOLOv5s model, the ODEL-YOLOv5s model has high robustness and realizes the accurate identification of obstacles in coal mine roadways in harsh environments, which also further verifies the validity of the improvement schemes used in this study.

To further validate the detection ability of the ODEL-YOLOv5s model, the images are collected in the Guqiao coal mine in Huainan City, Anhui Province, and a test set of 1000 images is established. The conventional YOLOv5s and ODEL-YOLOv5s models are tested on the 1000 images, respectively, and the total number of obstacles detected by the two models is counted. The detection results are shown in Table 4. The total number of obstacles contained in the 1000 test set images is 4212, and the total number of obstacles correctly detected by the conventional YOLOv5s is 4015, with 35 false detections and 162 missed detections, while the ODEL-YOLOv5 correctly detects 4186 obstacles, with 8 false detections and 18 missed detections. The detection results show that the number of correct detections in the ODEL-YOLOv5s model is 171 more than in the conventional YOLOv5s. The number of false detections and missed detections is 27 and 144 less than the conventional YOLOv5s, respectively, which validates that the proposed ODEL-YOLOv5s model still has high robustness in other coal mine roadway scenarios.

**Table 4:** Comparative experimental results in another coal mine.

Model	Correct detection	False detection	Missed detection	Total number
YOLOv5s	4015	35	162	4212
ODEL-YOLOv5s	4186	8	18	4212

## Conclusion

The obstacle identification and detection of existing driverless electric locomotives in the harsh environment of coal mine roadways is subject to the problems of low detection accuracy and slow speed. In accordance with the characteristics of obstacles in coal mine roadways, the ODEL-YOLOv5s model is proposed for obstacle detection in coal mine driverless electric locomotives. The main conclusions are as follows: (1) Increasing the diversity of obstacle features by image augmentation processing on the self-made dataset can effectively increase the detection accuracy of the model. (2) Introducing the CBAM attention mechanism in the model to redistribute the weights of target and non-target regions in the image makes the model more focused on the obstacle information, thus further increasing the detection accuracy. (3) Adding a small target detection layer in the model to further integrate the shallow features with the deep features, which effectively facilitates the detection of small obstacles in the model. (4) By optimizing the loss function and the non-maximum suppression method, the regression accuracy of the prediction boxes is improved, and the redundancy of the prediction boxes is effectively reduced. (5) The mAP of the proposed ODEL-YOLOv5s model is increased from 95.2 to 98.9% compared to the conventional YOLOv5s; the AP of small obstacle rock is increased from 89.2 to 97.9%; the detection speed of the model is 60.2 FPS; and the weight size and computation amount are 15.9 Mb and 27.6 GFLOPs, respectively. Compared with the existing mainstream target detection algorithms, the ODEL-YOLOv5s model shows the advantages of high detection accuracy, fast detection speed, and small model memory, which can effectively ensure the safe operation of coal mine driverless electric locomotives.

## Data Availability

The datasets generated and analyzed during the current study are available from the corresponding author on reasonable request.
